# Chaos in the Equilibrium: A Rare Complication of Dialysis

**DOI:** 10.7759/cureus.110596

**Published:** 2026-06-10

**Authors:** Rutuja Challawar, Jillianne Unas, Sai Shahane, Patricia Perez de Tagle, Sankalp Acharya, Priya Angi

**Affiliations:** 1 Internal Medicine, Monmouth Medical Center, Long Branch, USA; 2 Geriatrics, Leon Hess Cancer Center, Monmouth Medical Center, Long Branch, USA

**Keywords:** cerebral edema, dialysis disequilibrium syndrome, hemodialysis, osmotic imbalance, renal replacement therapy

## Abstract

Dialysis disequilibrium syndrome (DDS) is a rare but potentially life-threatening neurologic complication associated with hemodialysis (HD), particularly during initial treatments in patients with severe uremia. It is thought to result from rapid osmotic shifts between the plasma and the central nervous system during dialysis. We present the case of a 36-year-old woman with stage V chronic kidney disease secondary to diffuse lupus nephritis who developed suspected DDS during her first HD session. She presented with acute-on-chronic renal failure, severe metabolic acidosis, a creatinine of 12.09 mg/dL, and a blood urea nitrogen (BUN) of 162 mg/dL. Within 30 minutes of HD initiation, the patient developed headache and nausea, followed by generalized shaking, lateral tongue biting, and postictal confusion approximately 15 minutes after treatment completion, concerning for a generalized tonic-clonic seizure. Neuroimaging and electroencephalography were unremarkable, and she returned rapidly to baseline mental status. HD was subsequently resumed with reduced blood flow and dialysate rates, which she tolerated without recurrence of symptoms, supporting the diagnosis of DDS. This case highlights the importance of recognizing DDS as a clinical diagnosis of exclusion in high-risk patients initiating dialysis. Prevention through gradual urea clearance and individualized dialysis prescriptions remains the cornerstone of management, particularly in patients with severe uremia and metabolic derangements.

## Introduction

Dialysis disequilibrium syndrome (DDS) is a rare but potentially life-threatening complication of hemodialysis (HD) characterized by a spectrum of neurological manifestations occurring during or shortly after treatment. First described in 1962 in a Lancet publication, DDS is primarily attributed to the rapid removal of solutes from the extracellular fluid (ECF) plasma compartment during HD, resulting in the development of an osmotic gradient between the plasma and brain tissue [1]. This gradient may promote cerebral edema and increased intracranial pressure, leading to neurological symptoms. DDS most commonly occurs in patients with markedly elevated plasma urea levels, particularly those with chronic kidney disease undergoing initial or aggressive HD. Clinical manifestations range from mild symptoms such as headache and nausea to severe complications, including seizures, coma, and death [2].

The true incidence of DDS remains unclear and is believed to be underreported, as milder or transient symptoms may go unrecognized, while only severe presentations such as seizures or altered mental status are consistently documented. DDS is rarely observed in other dialysis modalities, such as continuous renal replacement therapy (CRRT), where solute and fluid shifts occur more gradually [3]. Here, we present a case of DDS in a patient with classic risk factors to increase clinical awareness and emphasize the importance of preventive strategies to reduce the risk of this complication.

## Case presentation

We describe a 36-year-old woman with stage V chronic kidney disease secondary to diffuse class IV lupus nephritis, rheumatoid arthritis, pulmonary hypertension, and systemic hypertension who presented with a seven-day history of upper respiratory symptoms, including a productive cough with whitish sputum, generalized weakness, chills, low-grade fever, nausea, vomiting, and poor oral intake. She denied chest pain, shortness of breath, abdominal pain, and changes in bowel habits. She reported preserved urine output and denied recent nonsteroidal anti-inflammatory drug (NSAID) use. On presentation, she was alert, oriented, and in no acute distress. Vital signs revealed a blood pressure of 159/109 mmHg, heart rate of 92 beats/min, respiratory rate of 20 breaths/min, a normal temperature, and oxygen saturation of 97% on room air.

Laboratory evaluation demonstrated low hemoglobin and an elevated white blood cell count. Creatinine and blood urea nitrogen (BUN) levels were markedly elevated relative to baseline, with a baseline creatinine of approximately 5 mg/dL. Bicarbonate was severely decreased, consistent with metabolic acidosis. Calcium was critically low, and potassium was mildly elevated (Tables 1, 2). A respiratory viral panel was positive for influenza A and B, and chest radiography was unremarkable.

**Table 1:** CMP on admission Baseline creatinine ~5 mg/dL CMP, comprehensive metabolic panel; A/G, albumin-to-globulin; ALP, alkaline phosphatase; eGFR, estimated glomerular filtration rate; AST, aspartate aminotransferase; ALT, alanine aminotransferase; BUN, blood urea nitrogen

Laboratory test	Value	Reference range	Interpretation
Sodium	139 mmol/L	135-145	Normal
Potassium	5.1 mmol/L	3.5-5.0	High
Chloride	108 mmol/L	98-110	Normal
Bicarbonate	<10 mmol/L	22-29	Critically low
BUN	162 mg/dL	7-20	Critically high
Creatinine	12.09 mg/dL	0.6-1.3	Critically high
eGFR	3.8 mL/min/1.73m²	>60	Critically low
AST	16 U/L	10-40	Normal
ALT	29 U/L	7-56	Normal
Albumin	3.8 g/dL	3.5-5.0	Normal
A/G Ratio	1.2	1.0-2.1	Normal
ALP	106 U/L	44-147	Normal
Calcium	4.9 mg/dL	8.5-10.5	Critically low
Total protein	6.9 g/dL	6.0-8.3	Normal

**Table 2:** CBC on admission CBC, complete blood count

Laboratory test	Value	Reference range	Interpretation
Hemoglobin	11.3 g/dL	12.0-16.0	Low
WBC	11.6 K/µL	4.0-11.0	High
Neutrophils	84%	40-70	High
Immature granulocytes	1%	0-0.5	High
Lymphocytes	11%	20-40	Low
Monocytes	4%	2-8	Normal
Eosinophils	0%	1-4	Low
Basophils	0%	0-1	Normal
Platelets	249 K/µL	150-450	Normal

She was admitted with acute-on-chronic kidney injury and influenza virus infection and was initially managed with 5% dextrose in water (D5W) and sodium bicarbonate infusion, prednisone for a possible lupus flare, and antiviral therapy with oseltamivir. Despite aggressive supportive care, her renal function showed minimal improvement, and urine output remained low, prompting the diagnosis of progression to end-stage renal disease.

**Figure 1:**
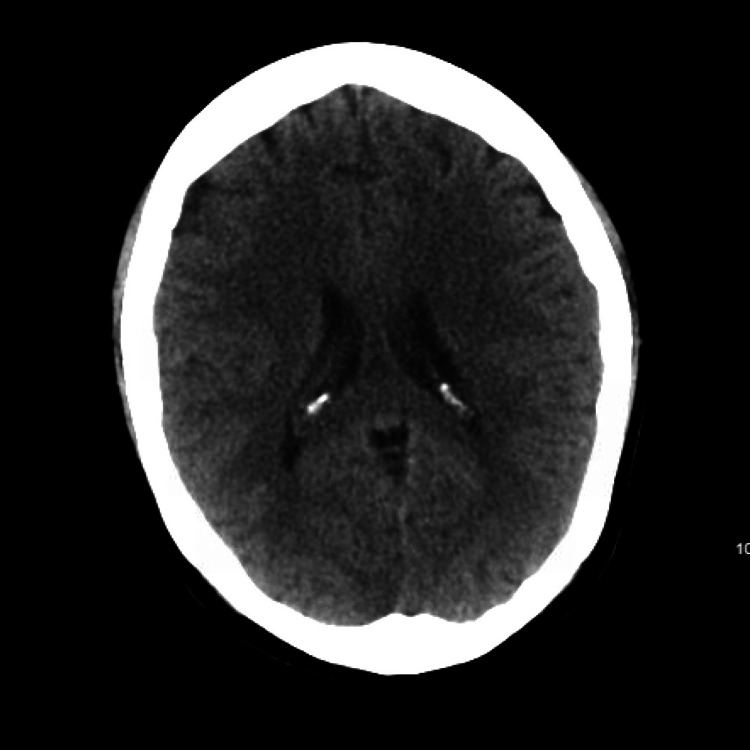
CT head obtained during the rapid response showing no acute abnormality of the brain parenchyma CT, computed tomography

HD was initiated via a newly placed central venous dialysis catheter. During the first dialysis session (150 minutes, a blood flow rate of 100-200 mL/min, ultrafiltration volume of 500 mL), the patient developed a headache and nausea. Approximately 15 minutes after treatment completion, she developed altered mental status, generalized shaking with arm twitching, and lip biting and lateral tongue biting. A rapid response was activated. The episode resolved spontaneously before the arrival of the response team; however, postictal confusion raised concern for a generalized tonic-clonic seizure. The patient rapidly returned to her baseline mental status. Neurology was consulted, and computed tomography (CT) of the head (Figure 1) and electroencephalography were unremarkable.

The following day, HD was resumed using a modified prescription designed to minimize rapid osmotic shifts, including no ultrafiltration, a higher dialysate potassium concentration, an increased dialysate calcium concentration, and a shorter treatment duration of 120 minutes. She tolerated the session without intradialytic or post-dialysis complications. Subsequent dialysis treatments were gradually advanced and remained uneventful, supporting DDS as the most likely etiology of the initial neurologic event. She was ultimately discharged with outpatient HD scheduled three times weekly, education regarding dialysis adherence, and follow-up at her primary clinic (Table 3).

**Table 3:** Timeline of clinical course CT, computed tomography; HD, hemodialysis

Time point	Clinical events
7 days before admission	Developed productive cough with whitish sputum, generalized weakness, chills, low-grade fever, nausea, vomiting, and poor oral intake
Day 0 (Admission)	Admitted with acute-on-chronic kidney injury in the setting of influenza A and B infection. Laboratory evaluation demonstrated severe azotemia, metabolic acidosis, hypocalcemia, and mild hyperkalemia. Initiated treatment with intravenous bicarbonate-containing fluids, prednisone, and oseltamivir
Hospital days 1-3	Persistent renal dysfunction with minimal improvement despite supportive therapy; progression to end-stage renal disease is suspected
HD session 1	Initiated HD via a central venous catheter (150 minutes, blood flow rate gradually increased from 100 to 200 mL/min, ultrafiltration volume of 500 mL). Developed headache and nausea during treatment
Approximately 15 minutes after session 1	Developed altered mental status, generalized shaking with arm twitching, and lip/tongue biting. Rapid response activated. Event resolved spontaneously but was followed by postictal confusion. Mental status returned to baseline shortly after
Post-event evaluation	Neurology consultation obtained. CT of the head and electroencephalography were unremarkable
HD session 2 (following day)	HD resumed with a modified prescription to minimize osmotic shifts (120 minutes, no ultrafiltration, higher dialysate potassium and calcium concentrations). No neurologic symptoms or complications occurred
Subsequent hospital course	Additional dialysis sessions were gradually advanced and well tolerated without recurrence of neurologic symptoms
Discharge	Discharged home with outpatient HD scheduled three times weekly, dialysis education, and outpatient follow-up

## Discussion

DDS is likely underreported due to its nonspecific presentation and the challenge of diagnosis, as it remains largely a clinical diagnosis of exclusion following dialysis initiation [4]. It most commonly occurs during or shortly after HD, as illustrated in this case, where the patient developed a tonic-clonic seizure during treatment. Early manifestations include headache, nausea, disorientation, restlessness, blurred vision, and asterixis, while severe cases may progress to confusion, seizures, coma, and death. Milder symptoms such as muscle cramps, anorexia, and dizziness may go unrecognized, contributing to underreporting [5].

Patients newly started on intermittent HD are at greatest risk, particularly those with markedly elevated blood urea levels (>60 mmol/L). Additional risk factors include severe metabolic acidosis, advanced age, chronic kidney disease, pediatric status, pre-existing neurologic disorders, and conditions associated with cerebral edema or increased blood-brain barrier permeability [6].

In this case, DDS likely resulted from a combination of a high BUN level, a first HD session, and severe metabolic acidosis. These factors outweighed the relatively lower risk typically associated with younger patients. The central pathophysiologic process involves the development of an osmotic gradient between the plasma and brain, leading to cerebral edema [7].

DDS has been recognized for over 50 years, although its exact pathophysiology remains incompletely understood. Two main mechanisms have been proposed. The first is the reverse urea effect, which occurs when rapid urea removal during HD outpaces its diffusion from the brain, creating a transient osmotic gradient that drives water into brain cells and causes cerebral edema; this effect is amplified in advanced kidney disease by increased aquaporin expression and reduced urea transporters [8-11]. The second is the intracerebral acidosis theory, which suggests that dialysis-induced reductions in intracellular pH, along with carbon dioxide retention [9,12], increase osmotic activity through electrolyte dissociation and the accumulation of organic osmolytes (e.g., glutamine, glutamate, taurine, and myoinositol), further contributing to cerebral edema [10,13].

Prevention of DDS is essential, particularly in high-risk patients, and focuses on minimizing rapid osmotic shifts. The cornerstone of management is gradual urea reduction over multiple sessions rather than aggressive single-session clearance. A practical target is an initial ~40% reduction in urea within the first two hours (Urea Reduction Ratio (URR) ≈ 0.4), with dialysis prescriptions individualized based on urea distribution volume and guided by kinetic modeling [14]. In practice, this involves starting with low blood flow rates (150-250 mL/min), smaller dialyzers, and shorter sessions (1-2 hours), followed by cautious escalation with close clinical monitoring [15].

Additional strategies aim to maintain osmotic stability and further reduce risk. Individualizing dialysate sodium can help limit fluctuations in plasma osmolality, particularly during early sessions in high-risk patients [4]. In those with significant fluid overload, initial ultrafiltration followed by shorter HD sessions may be used. CRRTs, such as continuous venovenous hemodiafiltration (CVVHDF), allow slower and more controlled solute removal. Patients with markedly elevated urea levels or pre-existing neurologic symptoms should be initiated on dialysis in an inpatient setting to enable close monitoring and gradual adjustment of therapy.

## Conclusions

DDS most commonly occurs in patients with end-stage renal disease who have severe uremia and other hyperosmolar features at the time of dialysis initiation. Younger individuals who have significant comorbidities are potentially at increased risk. Identifying and addressing multiple risk factors is therefore essential, emphasizing the need for individualized treatment strategies and close clinical monitoring in vulnerable patients.

Prevention remains the cornerstone of management, particularly when initiating HD. In the absence of standardized guidelines, the primary goal is to avoid rapid osmotic shifts by ensuring gradual urea clearance, allowing serum levels to decline progressively over several days rather than abruptly in a single session.
